# The Effects of Vitamin D-Enriched Mushrooms and Vitamin D3 on Cognitive Performance and Mood in Healthy Elderly Adults: A Randomised, Double-Blinded, Placebo-Controlled Trial

**DOI:** 10.3390/nu12123847

**Published:** 2020-12-16

**Authors:** Ian T. Zajac, Mary Barnes, Paul Cavuoto, Gary Wittert, Manny Noakes

**Affiliations:** 1 Commonwealth Scientific and Industrial Research Organisation—Health and Biosecurity, P.O. Box 10041, Adelaide 5000, Australia; cavuotop@gmail.com (P.C.); mannynoakes@icloud.com (M.N.); 2 Centre for Epidemiology and Biostatistics, Flinders University, Adelaide 5001, Australia; mary.barnes@flinders.edu.au; 3 Freemasons Foundation Centre for Men’s Health, North Terrace, Adelaide 5000, Australia; gary.wittert@adelaide.edu.au; 4 Centre for Nutrition and Gastrointestinal Disease, South Australian Health and Medical Research Institute, Adelaide 5000, Australia

**Keywords:** vitamin D, mushroom, cognitive function, mood

## Abstract

Despite abundant cross-sectional evidence that low vitamin D status is associated with risk of cognitive decline in ageing, interventional evidence for benefits of vitamin D supplementation is lacking. This study was a 6 month randomised, double-blinded placebo-controlled clinical trial of the effects of vitamin D3 (D3), enhanced vitamin D2 in a mushroom matrix (D2M), standard mushroom (SM) and placebo (PL) on cognition and mood in *n* = 436 healthy older male (49%) and female volunteers aged ≥ 60 years. Primary end points were change in serum vitamin D metabolites (25-OH-D, 25-OH-D2 and 25-OH-D3), cognitive performance, and mood over 24 weeks. Levels of total 25-OH-D and 25-OH-D3 were maintained in the D3 arm but decreased significantly (*p* < 0.05) in the remaining arms (D2M, SM and PL). Analysis also revealed differential changes in these metabolites depending on total vitamin D status at baseline. There were no significant effects of treatment on any of the measures of cognitive function or mood. Overall, the results show that daily supplementation of ~600 IU of vitamin D3 was sufficient to maintain 25-OH-D throughout winter months, but in contrast to existing cross-sectional studies there was no support for benefit of vitamin D supplementation for mood or cognition in healthy elderly people.

## 1. Introduction

Cognitive decline is observed throughout the ageing process. It is characterised by diminishing capacity in a variety of brain functions [1]. These age related declines in cognitive function are normal [2], even in the absence of underlying pathology [3] and they are most pronounced for tasks relying on information processing speed and executive functions [3,4]. Furthermore, this decline is not unexpected, with research showing that approximately 50% of older adults report concerns about memory function and that, for many, this is a most-dreaded component of ageing [5].

Vitamin D, by direct and indirect regulation of more than 200 genes, exerts bioactivity as a hormone and plays an important role in calcium absorption, tissue and immune cell growth, and inflammation [6]. The importance of vitamin D for brain health is supported by the presence of the enzyme that produces its active form—1α-hydroxylase—in cerebrospinal fluid [7]. Furthermore, the receptor for the active metabolite—1,25-dihydroxy vitamin D3—is found throughout the human brain [8]. Vitamin D has therefore been linked with neuron growth, differentiation and survival by its regulation of factors such as glutathione, growth factors, neurotrophins and neurotransmitters and to buffer calcium in the brain [9].

Humans obtain vitamin D from environmental and dietary sources. Vitamin D3 can be efficiently produced from sunlight (UV-B) exposure to human skin and moderate doses are available from consuming oily fish, egg yolk, meat and fortified foods [6]. However, these dietary sources provide <10% of the daily vitamin D requirement [10]. In comparison, vitamin D2, produced by UV conversion of ergosterol in fungi and plants, particularly mushrooms, represents a new, convenient and accessible source of dietary vitamin D2 [11]. The bioavailability of mushroom vitamin D2 and its capacity to contribute to vitamin D status has been substantiated with doses of 400 IU to 2000 IU/day, taken over 4 weeks to 3 months, producing increases in 25-OH-D2 and overall increases in total 25-OH-D [12,13,14,15].

Multiple systematic reviews and meta-analyses comparing cognitively normal with impaired, including Alzheimer Disease groups, have reported positive associations between vitamin D status and cognitive function in older adults [16,17,18,19,20,21]. For example, a recent meta-analysis of cross-sectional data demonstrated that low vitamin D status predicted executive dysfunctions including impaired mental shifting, information updating and processing speed [22]. However, like cognitive decline and dementia, vitamin D deficiency risk and deficiency per se is also related to ageing. Various age-related risk factors include inadequate dietary intake, inadequate exposure to sunlight, impaired transdermal and intestinal absorption, impaired metabolism in the liver and kidneys and medications that promote catabolism [23]. Therefore, the potential exists that the observed relationship between vitamin D and cognitive function is not causal but rather due to extraneous ageing-related variables.

To date, intervention studies exploring a causal link between vitamin D and cognitive function are lacking. Post-hoc analysis of data from the Women’s Health Initiative study explored incidence of probable dementia and mild cognitive disorder in women aged >65 years who were dementia free at baseline, and treated with either placebo or daily doses of calcium (1000 mg of calcium carbonate) plus vitamin D3—400 IU [24]. Results of these analyses indicated no difference in onset of dementia between the treatments. Elsewhere, in relation to mood states, a recent systematic review of effects of vitamin D supplementation on depressive symptoms indicated that a moderate, statistically significant benefit was evident for improving mood but only in those with clinical depression at the outset [25].

This clinical trial sought to evaluate whether vitamin D supplementation delivered either as enhanced vitamin D2 (UV-exposed mushroom, D2M) or D3 (synthetic) is causally related to cognition and mood in a healthy, cognitively asymptomatic, elderly cohort. We hypothesised that consumption of either D2 or D3 treatments would lead to an elevation in vitamin D after 24 weeks compared to standard mushroom (SM) and placebo (PL) controls. Furthermore, we hypothesised that D2 and D3 supplementation would benefit cognitive function and mood after 6 months of intervention. This article reports on the primary outcomes of this trial.

## 2. Materials and Methods

### 2.1. Study Design

This was a single-centre, double-blinded, placebo-controlled, 4-armed parallel-group randomised clinical trial. This study (http://www.anzctr.org.au; ACTRN12613000891729) was conducted at the Commonwealth Scientific and Industrial Research Organization (CSIRO) Clinical Nutrition and Health Research Clinic, Adelaide, South Australia from April through to October in both 2014 and again in 2015 (two cohorts). This study was conducted according to the ethical guidelines of the National Health and Medical Research Council of Australia. Approval to undertake this study was obtained from the CSIRO Human Ethics Committee and written informed consent was obtained from all participants. The complete protocol for the current randomised controlled trial has been published previously [26].

### 2.2. Participants and Screening

**Figure 1:**
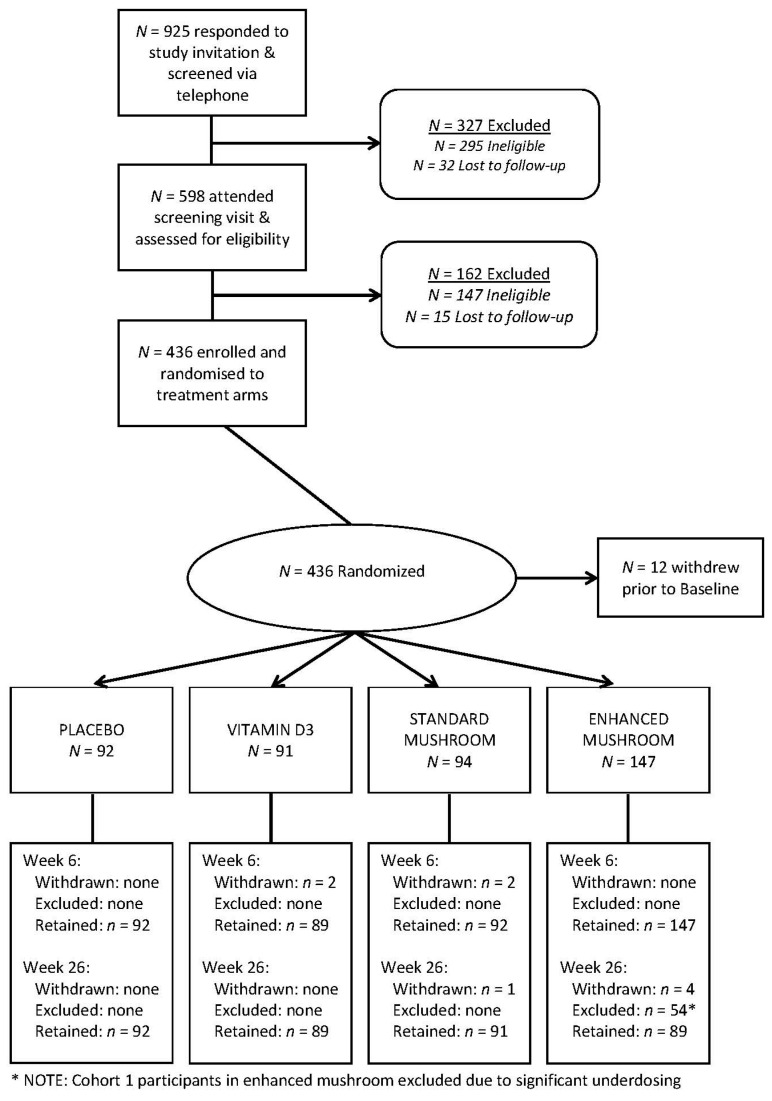
Flow of participants through the trial.

Community dwelling healthy elderly participants were recruited through a variety of study advertisements including newsletters, volunteer databases and radio. For logistic reasons, participants were recruited in two separate cohorts in 2014 and 2015, respectively. A total of *n* = 436 participants were randomised to trial arms and a consort diagram showing progression through screening and recruitment is provided as Figure 1. Inclusion and exclusion criteria were assessed at screening through a variety of clinical measures including our standard medical questionnaire, the Centre for Epidemiological Studies-Depression Scale (CES-D), the Obstructive Sleep Apnoea 50 questionnaire (OSA−50) [27], and the Mini-Mental State Examination (MMSE) [28].

Inclusion criteria were: healthy males and females aged 60–90 years of age; fluent in the English language (for valid completion of the cognitive test battery); not taking any form of vitamin D supplementation for at least three months prior to this study and willing to refrain from additional supplementation during this study.

Exclusion criteria were: inability to swallow tablets; physical inability to attend our research clinic; poor cognitive function (score ≤ 24 on the MMSE); depression (scores ≥ 16 on the CES-D); diagnosis of intellectual disability, dementia, or neurological disorder including but not limited to cerebral vascular disease; previous head injury, stroke, or coronary artery bypass or neurosurgical procedure; history of smoking, alcohol or drug abuse; metabolic disease including diabetes; untreated asthma; shift workers; people who habitually sleep < 6 h per night; untreated but probable obstructive sleep apnoea (OSA−50 responses assessed by study medical doctor); abnormal thyroid function; abnormal vitamin B12 status; history of psychosis and/or taking anti-psychotic medication; or history of epilepsy and/or taking anti-epileptic medication; kidney disease or kidney function impairment, and/or a gastro-intestinal condition that interferes with nutrient absorption; sensitivity to or intolerance for consuming mushrooms. Figure 1 shows participant flow throughout this study.

### 2.3. Intervention

Participants were randomised by an independent statistician into four treatment groups using a computer-based minimization process to assist with balancing groups for sex and age: (1) vitamin D2-enriched mushroom (D2M); (2) vitamin D3 (D3); (3) standard mushroom (SM); or (4) placebo (PL). The four study arms were stratified with regards to age and gender. Participants attended the CSIRO clinic at 3 time points: baseline, 5 weeks and 24 weeks (~6 months). At each clinic visit, participants completed the cognitive assessment tasks, mood questionnaires and provided blood samples at baseline and end point. Participants were instructed to commence consumption of two capsules daily on the day following their baseline visit. Participants were able to consume the capsules at any time during the day and returned unused capsules at the end of the intervention period as a measure of compliance.

Capsules for all arms were provided to the research clinic in opaque containers marked with an allocation number to ensure blinding of research staff and also participants. The active intervention capsules were intended to provide a total daily dosage of 600 IU of either D2 (D2M) or D3 across two capsules. Mushroom powder for the D2M and SM groups was prepared as described previously [26]. Briefly, this involved freeze drying and grinding mushrooms to ensure suitable moisture (<5%) and water activity (<0.065) levels. In order to boost levels in the D2M arm, batches of mushroom powder were exposed to UV-B light, driving the conversion of ergosterol to vitamin D2, before vacuum sealing and storage at 4 °C, using a treatment process fully described elsewhere [26,29]. All capsules were manufactured with a shelf life of 2 years for storage at 4 °C and single batches of each treatment used for years 1 and 2 of the clinical study.

### 2.4. Cognition and Mood Measures

Cognitive function was assessed using the CSIRO Cognitive Assessment Battery (C-CAB) (Commonwealth Scientific and Industrial Research Organization, Adelaide, South Australia) as described previously [26]. Briefly, the C-CAB measures performance levels for a range of distinct cognitive abilities including processing speed, reaction time/attention, speed of reasoning, speed of memory scanning, verbal working memory, spatial working memory, recognition memory, and overall quality of memory. Abilities measured in the C-CAB are those considered most susceptible to change and which are commonly included in batteries designed to measure the impact of nutraceuticals on cognitive performance [30,31]. The methods and procedures underpinning the tasks have been used in previous studies conducted by current authors [32,33] and have been fully described previously in the published protocol [26]. Standardised questionnaires were used to assess mood and depressive symptoms at each of the three clinic visits. These measures included the Positive and Negative Affect Schedule (PANAS) [34], the 21 item Depression Anxiety and Stress Scales (DASS−21) [35], and the General Happiness Scale [36].

### 2.5. Vitamin D Metabolite and Serum Biomarkers

Vitamin D metabolite levels were analysed by an accredited pathology laboratory (SA Pathology, Adelaide, Australia). Levels of 25-hydroxy vitamin D2 and 25-hydroxy vitamin D3 were measured in blood sera by an accredited pathology laboratory (SA Pathology, Adelaide, Australia) using high throughput liquid chromatography tandem mass spectroscopy (LC-MSMS) [37]. As we have described elsewhere [32] this method involved precipitation of serum proteins with acetonitrile, with addition of internal standard (25- Hydroxyvitamin D3 26,26,26,27,27,27-d6, Medical Isotopes Inc, Pelham, NH, USA). Calibration was conducted using serum-based standards (Recipe Chemicals, Munich, Germany) traceable to NISTSRM972 (Gaithersburg, MD, USA). Chromatographic analysis was conducted using a Turboflow C18XL column (Thermo Scientific, Scoresby, Victoria, Australia) for on-line sample clean up, followed by an Accucore C18 analytical column (50 × 3 mm, Thermo Scientific) on a TLX4 multiplexed chromatography system (Thermo Scientific). Mass spectrometry detection utilised a TSQ Quantum Access MAX (Thermo Scientific) with APCI ionization in the positive mode and monitoring 3 MRM transitions per analyte. The limit of quantitation of the method was 5.2 and 5.0 nM for 25-OH vitamin D3 and 25-OH vitamin D2, respectively. Accuracy was assessed by running 20 samples from 4 cycles of the DEQAS external quality assurance program (Charing Cross Hospital, London, UK) for which the median value obtained by all LC-MS laboratories was between 90 and 109%, with a mean of 98%. The average coefficient of variation for these analysis was 7.5%.

### 2.6. Anthropometric and Covariate Measures

Height was measured using a stadiometer (SECA, Hamburg, Germany) and body weight using calibrated electronic digital scales (A & D Mercury HW-PW200 Platform Scales, A & D Australasia, Thomastown, Australasia). The contribution of environmental intakes of vitamin D3 was also assessed via a validated 7 day recall questionnaire completed by participants at each of the 3 clinic visits, in which both exposure time and skin type was recorded [38]. This survey was used to assess equivalence of the cohorts in terms of participant’s exposure to an environmental source of vitamin D.

### 2.7. Power and Statistical Analysis

Power analyses were based on the Multivariate Repeated-Measures ANOVA (MANOVA) procedure and conducted using G*Power v3.1.7 (Heinrich Heine University, Düsseldorf, Germany). Recruitment of 100 participants per group was calculated to yield power of 95% to detect a small but statistically significant time × treatment effect size of f = 0.15. Accounting for a high drop-out rate of 25%, there would remain 80% power to detect an interaction effect size of f = 0.15. In the event of significant main or interaction effects, post-hoc pair-wise comparisons would be performed. With *n* = 100 per group, there would be 80% power to detect an effect size of 0.4 (Cohen’s d) between any two groups. Allowing for attrition (~25%), there would remain 80% power to detect a medium effect size of ~0.45 (Cohen’s d).

All statistical analysis was conducted using SPSS software version 23 (IBM Corporation, New York, USA). Univariate outliers of cognition and mood data (scores greater than 3.5 standard deviations (SD) from their respective means) were reduced to the next highest/lowest score within the main body of the distribution, in order to reduce their influence in analyses. Then, transformations of latency derived measures were undertaken to normalize distributions of the cognitive tasks; in line with previous studies (Danthiir, Wilhelm, Schulze, & Roberts, 2005) work rates representing the number of items correctly completed per second were calculated for all measures except for the overall quality of memory domain, which is a composite accuracy-only measure. These work rates were then weighted by accuracy (WR × % correct on respective task) to control for any speed–accuracy trade offs. Finally, domain scores were calculated by averaging performance across the relevant indicator tasks described previously [26].

Intention-to-treat analyses were undertaken and included all participants that attended for baseline, whether or not they withdrew before follow up, but excluding the 2014 cohort D2M participants who were underdosed (see explanation in results section below). For the measure of 25-OH-D2, participants were classified as ≥5 nM (no/yes) at each time point and, given the binary nature of this variable, it was modelled using Mixed-Effects Logistic Regression. Total 25-OH-D and 25-OH-D3 outcomes were modelled using Linear Mixed-Effects Regression (LMER) models. The repeated nature of the measurements were accounted for by permitting random slopes and intercepts for each participant and fixed effects (time, treatment and time × treatment) were specified. For cognitive and mood outcomes, a random slope for time was modelled enabling the comparison of rate of change on these measures over the three study visits. In the event of significant main and interaction effects, sub-groups defined by sex, and vitamin D adequacy at baseline (cut off of ≥75 nM [39]) were further examined by incorporating a three-way interaction (time × treatment × subgroup) and the significance of these were considered relative a conservatively modified *p* ≤ 0.025 criterion to account for the multiple comparisons conducted herein. However, these subgroup analyses were not undertaken for the 25-OH-D2 groups because of low overall cell counts. An unstructured covariance matrix was specified for all random effects and all models controlled for age and BMI at baseline, sex and cohort, with the addition of MMSE screening scores and APOE-ε4 status (yes/no) for cognitive and mood models. The assumption of normally distributed residuals was assessed and was satisfied for all models.

## 3. Results

### 3.1. Capsule Composition, Compliance and Adverse Events

Compliance of all participants who attended the end-point visit exceeded 95%, with no serious adverse events reported. Analysis of vitamin D present in the intervention capsules was performed at the end of each cohort. Testing of the intervention product at the end of 2014 (Cohort 1) revealed that the D2M group had received a significantly lower dosage than intended due to a manufacturing error. Participants received only 114 IU per day (57 IU per capsule) as opposed to the target dosage of 600 IU. Therefore, the batch of capsules used in the second cohort was assessed prior to commencement of the 2015 cohort (500.2 IU per capsule) and again at the end point (500.2 IU per capsule) and was found to be stable and in excess of the daily target of 600 IU. Analysis of levels in the D3 arm for 2014 (367.2 IU per capsule) and 2015 (374.4 IU per capsule) were found to be acceptable and slightly higher than the target 600 IU daily dosage. Given the underdosing of D2M participants in 2014, they were excluded from the trial and all analyses.

### 3.2. Baseline Characteristics

Three-hundred and seventy remaining participants completed a baseline assessment and formed the intention-to-treat sample for main-effects analyses. Characteristics of these participants are presented in Table 1. The groups were relatively balanced although there was evidence of some differences for BMI: participants in the SM group had a lower BMI than in the D3 and PL groups (both *p* < 0.02). Analysis of exposure to environmental vitamin D using sun exposure scores controlling for sex, age and cohort, showed no statistically significant differences between the treatments groups over the three study visits in terms of their sun exposure (*p* = 0.57). However, as expected due to this study coinciding with winter months, the overall mean sun exposure was lower at visit two than baseline and end-point visits (*p* < 0.05).

### 3.3. Vitamin D Outcomes

**Figure 2:**
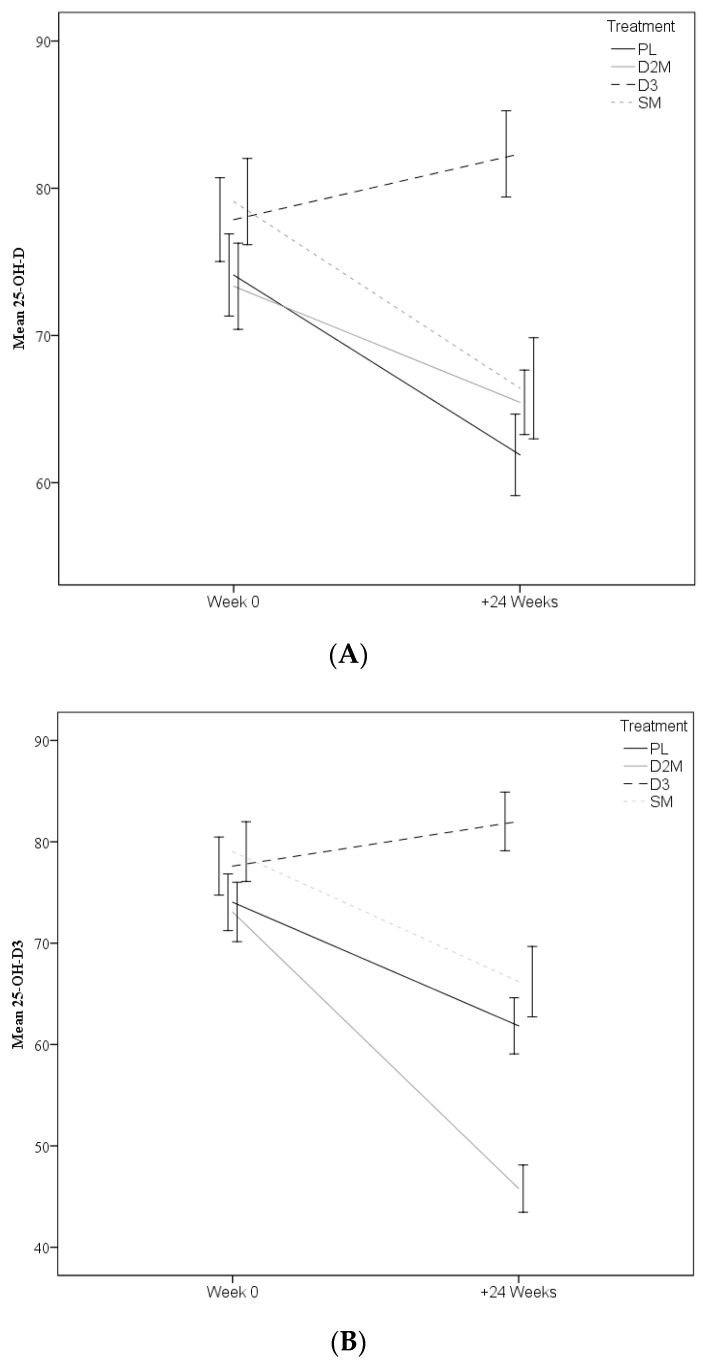
Changes in 25-OH-D (**A**) and 25-OH-D3 (**B**) per treatment arm. Error bars represent ±1.5 SE. PL = Placebo, D2M = enhanced vitamin D2 in mushroom, D3 = Vitamin D3, SM = Standard Mushroom.

In individuals retained through to the final visit, average adherence to treatment was assessed on the basis of returned capsules. Adherence was high—89% (D2M), 89% (D3), 91% (SM) and 90% (PL)—and not different across treatment arms (one-way ANOVA, *p* = 0.48). Despite this, the models revealed significant time, treatment and interaction effects (see Table 2 and Figure 2). Overall, mean levels of total 25-OH-D declined over 24 weeks (all *p* ≤ 0.01). However, the interaction effect showed that compared to PL, the decline in 25-OH-D in the D2M arm was less negative and slower (*p* = 0.047), whilst the change was actually positive for D3 (*p* < 0.001). Similarly, 25-OH-D3 levels declined overall and the interaction effect indicated that compared to placebo, the decline in D2M was more pronounced and faster (*p* < 0.001), whilst change was somewhat more positive in D3 (*p* < 0.001). The PL and SM treatments were not different for either of these measures. As shown in Table 2, levels of 25-OH-D2 were generally not detectable at baseline in any group. However, the number of individuals with detectable levels (>5.0 nM) increased with the interaction term, demonstrating that this was only apparent for the D2M arm (*p* < 0.001).

**Figure 3:**
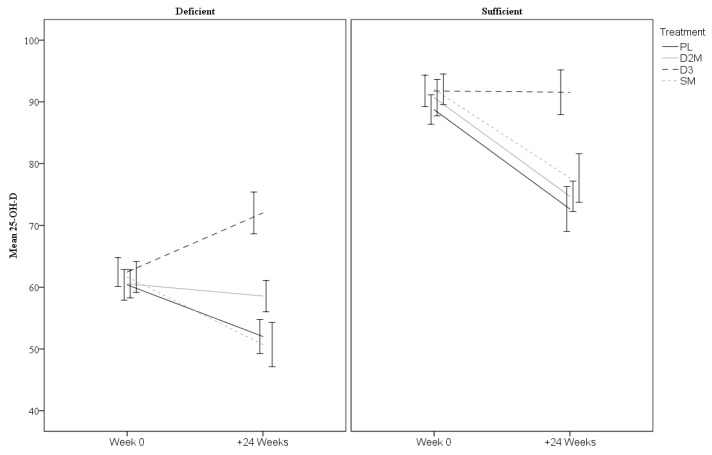
Changes in 25-OH-D per treatment arms for baseline total 25-OH-D-deficient (<75 nM) and -sufficient subgroups. Error bars represent ±1.5 SE. D2M (enhanced D2 mushroom), D3 (vitamin D3); SM (standard mushroom), and PL (placebo).

**Figure 4:**
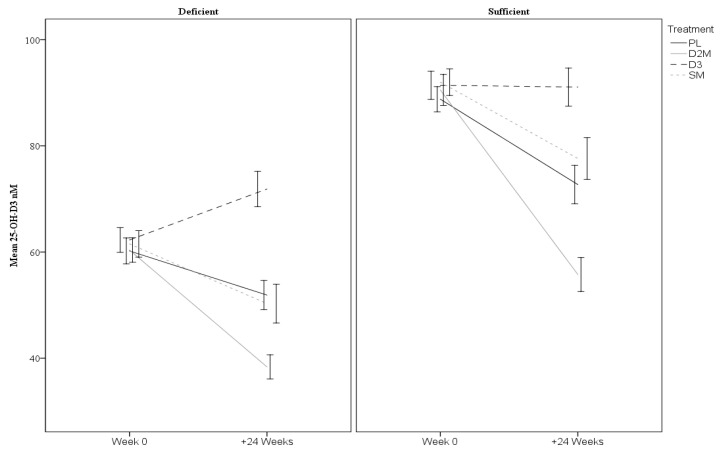
Changes in 25-OH-D3 per treatment arms for baseline total 25-OH-D-deficient (<75 nM) and -sufficient subgroups. Error bars represent ± 1.5 SE. D2M (enhanced D2 mushroom), D3 (vitamin D3); SM (standard mushroom), and PL (placebo).

Further planned analysis of sex and baseline vitamin D subgroups were undertaken by including three-way interactions (time × treatment × subgroups). The descriptive statistics for sex and baseline vitamin D subgroups are provided in Table 3. The three-way interaction for sex was not statistically significant indicating no differential effects across males and females for total 25-OH-D (*p* = 0.66) or 25-OH-D3 (*p* = 0.52). However, there was a three-way interaction with baseline vitamin D status for 25-OH-D (*p* < 0.001). Comparisons indicated that the degree of change differed between subgroups for each treatment level (all *p* ≤ 0.04). As shown in Figure 3, 25-OH-D increased in the D3 arm but only in participants who were deficient at baseline (it remained relatively stable in sufficient individuals). The rate of 25-OH-D decline within the remaining treatments differed depending on baseline status with faster declines in the sufficient groups. The three-way interaction for 25-OH-D3 was also significant (*p* < 0.001) with comparisons indicating that degree of change differed between subgroups for D2M, D3 and PL treatments (all *p* ≤ 0.001), but not for SM (*p* = 0.07). Quite different trajectories of change were apparent across the groups as shown in Figure 4, with faster rates of decline notable for the D2M treatment.

### 3.4. Cognitive and Mood Outcomes

Results of models assessing cognitive outcomes are provided in Table 4. Overall time effects showed that for all but the quality of memory domain, participant’s performance improved from baseline to end point, which is common across repeated cognitive assessments. There was only one interaction effect which was for the verbal working memory domain (*p* = 0.04). This showed that the slope of performance across the three visits was slightly negative for D3 and significantly different from the slope for the placebo group (*p* = 0.009), which was positive (i.e., performance improved over time). The slope for D2M and SM conditions was not different from PL, indicating that performance in these groups increased at a similar rate to PL and these increases are therefore not attributable to active intervention. None of the three-way interactions with sex and vitamin D subgroups exceed the adjusted criteria for significance (see statistical analysis section; descriptive data are not presented for brevity).

Results of models assessing mood outcomes are provided as Table 5. There were overall time effects for stress and negative affect showing decreases in these constructs over time in the combined sample. However, there were no significant treatment or time × treatment interaction effects for any mood outcome measures. Of all mood outcomes, only the three-way interaction of happiness with baseline vitamin D level exceeded criteria (*p* = 0.009). In this model, slopes were different between vitamin D-deficient and -sufficient subgroups for PL (increasing only in the deficient group, *p* < 0.01) and SM groups (decrease in the deficient yet increase in the sufficient group, *p* = 0.03); descriptive data for these models are not presented for brevity.

## 4. Discussion

This paper reports on the first randomised trial to explore a causative association between vitamin D supplementation and cognitive function in healthy community dwelling older adults. Participants in this trial received either vitamin D3 or enhanced D2 in a mushroom matrix for 24 weeks, with dosing levels approximating the recommended daily adult requirement of 600 to 800 IU [10]. The active interventions were shown to differentially impact vitamin D status through the winter months of this trial. Contrary to the hypothesis, neither the D2M nor D3 treatments resulted in elevated total vitamin D levels after 24 weeks. However, the D3 treatment was effective at maintaining vitamin D levels, with no significant declines observed for this group overall. Contrary to this, total vitamin D in participants receiving the D2 enhanced mushroom significantly declined. This decline was apparent despite the delivery of a notably higher (1000 IU) than anticipated (600 IU) dosage of D2, which exceeded the RDA [10] and was contrary to expectations. The present study is unable to glean the reasons for this but it may reflect either or both of physiological or analytical factors. Levels of 25-OH-D3 were also found to decline significantly faster in the D2M treatment compared to placebo (Figure 1).

Despite these main findings, differences were evident in rate of change of vitamin D according to baseline vitamin D status. In particular, it was noted that total 25-OH-D and 25-OH-D3 levels increased significantly in deficient individuals assigned to the D3 treatment, but these same measures remained stable in those sufficient at baseline. However, in the D2M arm, total 25-OH-D levels remained reasonably stable but only in individuals who were deficient at baseline. For 25-OH-D3, more rapid declines were evident in the D2 treatment than in the PL condition, and arguably faster in individuals sufficient in D3. This finding supports previous reports of differentiated effects of vitamin D2 versus D3 on circulating 25-OH-D, which consistently show that supplementation with vitamin D2 induces downward adjustment of the level of 25-OH-D3 proportional to the increase in 25-OH-D2 [14,40,41].

Although previous evidence of a relationship between vitamin D and cognitive status is apparent in existing literature [16,17,18,19,20,21], with lower levels associated with poor functional state, this study has shown no evidence of benefit of vitamin D supplementation across a broad range of cognitive domains assessing speed, reasoning and memory throughout winter months. The time effects noted were present overall and were not due to treatment. Thus, they are interpreted as practice effects which are common in clinical trials with repeated cognitive assessments [42]. Although no effects of treatment with either D2M or D3 were found in terms of their affecting cognitive function, this result might not be considered unusual given that total vitamin D levels declined. Even in the D3 treatment, levels did not increase other than in deficient individuals. These results cannot discount the potential benefits of higher vitamin D doses and further research should consider the impact of different dosage levels for cognitive benefit.

In addition to cognitive function, we explored a range of mood outcomes including anxiety, stress, depression, positive and negative affect, and general happiness. Again, there were no results reflecting differential impacts of active treatments over time. These results are in contrast to some prior studies demonstrating efficacy of vitamin D for improving measures of depression in non-depressed [43] and depressed individuals [44]. The present study sample comprised of healthy adults free from depression. Therefore, their capacity to experience positive changes in mood is compromised. In line with a recent meta-analysis, these results support the conclusion that vitamin D supplementation is ineffective in non-depressed persons [25].

### Study Limitations and Strengths

This study had several limitations. First, this was a single-centre study that utilised a relatively short treatment duration. It has been suggested that studies examining active interventions in terms of their exerting cognitive benefits should utilise durations as long as 5 years in otherwise healthy adults [45], whom formed the basis of the present study. It has been recommended therefore that future research might benefit from targeting individuals at risk of enhanced rates of decline [45], such as those exhibiting age-associated memory impairment in the absence of any underlying pathology [45]. Finding the right balance between conducting heavily controlled trials in healthy adults as opposed to studying those at greater risk, for whom myriad confounding factors are likely to exert influence, is a challenge for future cognitive interventions.

Second, this study did not control for vitamin D status at baseline and stratify randomisation accordingly. In the present case, this approach was adopted in order to avoid the necessity to refer-for-treatment individuals who were deficient in vitamin D but whom might benefit most from the active intervention. Nevertheless, it resulted in the presence of notable differences between the intervention arms in terms of overall vitamin D status which should be controlled for in future studies. The overall dosage used (~700 ID of D3, or 1000 IU of D2) might also be considered suboptimal for exerting benefits in otherwise healthy individuals. For example, studies that have shown benefits of vitamin D for improving mood have used as high as two-to-four times the dosage used in this trial [43,44].

Despite these limitations, there were distinct strengths to this study. This trial represents the first highly controlled randomised clinical trial assessing the efficacy of vitamin D supplementation for cognitive, mood and metabolic benefit in healthy community dwelling adults aged ≥60 years throughout winter months, when environmental vitamin D exposure is reduced. Participants were heavily screened to exclude a broad range of confounding factors including compromised cognitive function and depression. Furthermore, the cognitive assessments incorporated a robust battery of tests measuring distinct cognitive domains (e.g., reaction time and speed of processing). The strength of this methodology is that task-specific variance is removed from the cognitive data thus enabling the analysis of true underlying cognitive abilities [33]. The measurement of mood also comprised a broad range of standardised measures and mood constructs including both positive and negative states. Thus, these findings should be considered robust and generalisable to generally healthy community dwelling older adults. Authors should discuss the results and how they can be interpreted in perspective of previous studies and of the working hypotheses. The findings and their implications should be discussed in the broadest context possible. Future research directions may also be highlighted.

## 5. Conclusions

This is the first clinical evaluation of effects of supplementation of vitamin D2-enhanced mushroom on cognition and mood in a healthy elderly cohort. The four treatment arms compared vitamin D2 mushroom, vitamin D3, standard mushroom and placebo. D2M treatment resulted in a significant increase in 25-OH-D2 but an overall decline in total vitamin D, which was effectively maintained in the D3 condition only. Further, there was no benefit of vitamin D2-enhanced mushroom or D3 treatment for measures of cognitive function or mood. Based on the findings presented herein, it can be concluded that vitamin D supplementation in otherwise healthy, elderly populations free from depression and diminished cognitive capacity is ineffective at least in terms of helping improve either of these outcomes (mood or cognition), at the dosages used herein. It must be acknowledged that this result may not generalise to other populations such as those deficient in vitamin D, or those with objective cognitive impairments. Supplementation with ~600 IU daily of D3 does, however, appear sufficient for maintaining total vitamin D levels through winter. Future studies in healthy adults should seek to augment doses of vitamin D and longer durations to determine any long-term benefits during ageing.

## Figures and Tables

**Table 1:** Characteristics of baseline participants by trial group.

VARIABLE	Vitamin D Enhanced Mushroom (*n* = 93)	Vitamin D3 (*n* = 91)	Standard Mushroom (*n* = 94)	Placebo (*n* = 92)
Female, %	51.6	53.8	51.1	51.1
APOE-ε4 0/1/2, *n*	72/21/0	72/17/2	76/18/0	72/18/2
Smoking Behaviour, *n*				
Smoker	0	1	0	0
Ex-Smoker	17	14	20	12
Never-Smoker	76	76	74	81
Alcohol Behaviour, *n*				
Regular	69	65	58	0
Rarely	7	2	2	67
Never	17	24	34	25
Mean Alcohol Consumption, L/week	1.5 (1.5)	1.2 (1.1)	1.1 (1.0)	1.3 (1.4)
Age in years	69.7 (6.6)	70.8 (6.4)	69.4 (6.7)	70.1 (5.7)
BMI *	27.5 (4.0)	28.0 (4.0)	26.6 (4.0)	28.2 (4.1)
MMSE Score	28.6 (1.3)	28.5 (1.4)	28.4 (1.5)	28.6 (1.4)
Mean Sun Exposure Score				
Week 0	21.4 (10.3)	21.6 (10.1)	21.0 (8.6)	21.5 (11.1)
+5 Weeks	15.4 (8.6)	16.4 (8.4)	16.0 (7.3)	17.8 (10.1)
+24 Weeks	20.1 (10.1)	22.9 (10.4)	20.0 (9.1)	22.8 (9.5)

Mean (SD), unless otherwise indicated. * One-way ANOVA (*p* = 0.03). APOE = Apolipoprotein E; *n* = number of participants; MMSE = Mini-Mental State Examination.

**Table 2:** Means (±SE) for 25-OH-D and 25-OH-D3 metabolite levels and proportion of 25-OH-D2 > 5.0 nM for treatment groups across study visits.

Measure	Visit	D2M		D3		SM		PL		Model *p*-Values
25-OH-D	Baseline	74.64	±2.08	77.80	±1.94	77.95	±1.92	74.13	±1.94	T *p* < 0.001 Tr *p* = 0.052 T × Tr *p* < 0.001
	+24 Weeks	66.76	±2.06	82.24	±1.90	64.91	±1.88	61.91	±1.89	
25-OH-D3	Baseline	74.30	±2.09	77.57	±1.95	77.91	±1.92	74.09	±1.95	T *p* < 0.001 Tr *p* < 0.01 T × Tr *p* < 0.001
	+24 Weeks	47.02	±2.07	81.95	±1.92	64.71	±1.90	61.90	±1.90	
25-OH-D2 > 5.0 nM	Baseline	3	(3.2%)	3	(3.3%)	1	(1.1%)	1	(1.1%)	T *p* < 0.001 Tr *p* < 0.001 T × Tr *p* < 0.001
	+24 Weeks	88	(94.6%)	4	(4.4%)	3	(3.2%)	3	(3.3%)	

D2M (enhanced D2 mushroom), D3 (vitamin D3); SM (standard mushroom), and PL (placebo). Models controlled for baseline age, sex, BMI and cohort. *p*-value for time *x* treatment interaction term in mixed-effects regression models.

**Table 3:** Means (±SE) for 25-OH-D and 25-OH-D3 sex and vitamin D subgroups.

VARIABLE			D2M		D3		SM		CONTROL	
Male, *n*			*48*		*42*		*46*		*45*	
	25-OHD	Baseline	75.92	±2.53	79.12	±2.87	79.11	±2.85	72.87	±2.49
		+24 Weeks	66.38	±1.92	84.67	±2.85	67.67	±3.7	60.47	±2.45
	25-OHD3	Baseline	75.57	±2.55	78.73	±2.89	78.97	±2.88	72.7	±2.5
		+24 Weeks	48.95	±2.02	84.14	±2.81	67.46	±3.75	60.34	±2.47
Female, *n*			*45*		*49*		*48*		*47*	
	25-OHD	Baseline	70.6	±2.97	76.8	±2.54	79.08	±2.71	75.3	±2.76
		+24 Weeks	64.39	±2.26	80.26	±2.67	65.2	±2.75	63.26	±2.76
	25-OHD3	Baseline	70.42	±2.96	76.64	±2.55	79.1	±2.7	75.32	±2.76
		+24 Weeks	42.09	±2.31	80.11	±2.65	64.98	±2.76	63.28	±2.75
Deficient, *n*			*54*		*44*		*40*		*47*	
	25-OHD	Baseline	60.55	±1.52	62.45	±1.57	61.65	±1.67	60.38	±1.65
		+24 Weeks	58.57	±1.69	72.02	±2.26	50.71	±2.39	52.02	±1.85
	25-OHD3	Baseline	60.38	±1.54	62.27	±1.55	61.53	±1.67	60.2	±1.63
		+24 Weeks	38.36	±1.52	71.88	±2.23	50.28	±2.43	51.89	±1.83
Sufficient, *n*			*39*		*47*		*54*		*45*	
	25-OHD	Baseline	90.69	±1.97	91.77	±1.7	92.02	±1.66	88.75	±1.59
		+24 Weeks	74.71	±1.65	91.55	±2.42	77.68	±2.61	72.66	±2.42
	25-OHD3	Baseline	90.52	±1.95	91.42	±1.77	92	±1.67	88.79	±1.58
		+24 Weeks	55.77	±2.14	91.07	±2.39	77.62	±2.62	72.7	±2.41

D2M (enhanced D2 mushroom), D3 (vitamin D3), SM (standard mushroom). Classified as 25-OH-D deficient (<75 nM) or sufficient at baseline. *n* = number of participants.

**Table 4:** Descriptive statistics (mean ± SE) for cognitive domain measures.

COGNITIVE MEASURE		D2M		D3		SM		PL		Model *p*
Reaction Time	Week 0	1.56	±0.03	1.55	±0.03	1.5	±0.03	1.55	±0.03	T *p* < 0.001
	+5 Weeks	1.58	±0.03	1.57	±0.04	1.57	±0.03	1.6	±0.03	Tr *p* = 0.65
	+24 Weeks	1.61	±0.03	1.61	±0.04	1.57	±0.03	1.59	±0.03	T × Tr *p* = 0.82
Speed of Processing	Week 0	0.49	±0.01	0.5	±0.01	0.47	±0.01	0.48	±0.01	T *p* < 0.001
	+5 Weeks	0.5	±0.01	0.51	±0.01	0.48	±0.01	0.5	±0.01	Tr *p* = 0.10
	+24 Weeks	0.5	±0.01	0.51	±0.01	0.48	±0.01	0.5	±0.01	T × Tr *p* = 0.51
Speed of Reasoning	Week 0	0.8	±0.02	0.79	±0.02	0.73	±0.02	0.79	±0.02	T *p* < 0.001
	+5 Weeks	0.85	±0.02	0.83	±0.03	0.77	±0.02	0.83	±0.02	Tr *p* = 0.21
	+24 Weeks	0.86	±0.03	0.86	±0.02	0.8	±0.02	0.83	±0.02	T × Tr *p* = 0.77
Speed of Memory Scanning	Week 0	0.75	±0.02	0.73	±0.02	0.7	±0.02	0.72	±0.02	T *p* < 0.001
	+5 Weeks	0.77	±0.02	0.75	±0.02	0.72	±0.02	0.75	±0.02	Tr *p* = 0.39
	+24 Weeks	0.77	±0.02	0.74	±0.02	0.74	±0.02	0.74	±0.02	T × Tr *p* = 0.47
Recognition Speed	Week 0	0.6	±0.02	0.61	±0.02	0.56	±0.01	0.59	±0.02	T *p* < 0.001
	+5 Weeks	0.6	±0.02	0.59	±0.02	0.56	±0.01	0.58	±0.02	Tr *p* = 0.39
	+24 Weeks	0.65	±0.02	0.63	±0.02	0.59	±0.02	0.63	±0.01	T × Tr *p* = 0.43
Verbal Working Memory	Week 0	0.79	±0.02	0.82	±0.02	0.74	±0.02	0.79	±0.02	T *p* = 0.06
	+5 Weeks	0.8	±0.02	0.83	±0.02	0.78	±0.02	0.83	±0.02	Tr *p* = 0.05
	+24 Weeks	0.82	±0.02	0.8	±0.02	0.76	±0.02	0.82	±0.02	T × Tr *p* = 0.04
Spatial Working Memory	Week 0	0.32	±0.02	0.29	±0.01	0.27	±0.01	0.3	±0.01	T *p* < 0.001
	+5 Weeks	0.29	±0.01	0.29	±0.01	0.28	±0.01	0.26	±0.01	Tr *p* = 0.32
	+24 Weeks	0.36	±0.02	0.33	±0.01	0.33	±0.01	0.33	±0.01	T × Tr *p* = 0.59
Overall Quality of Memory Performance	Week 0	0.79	±0.01	0.78	±0.01	0.76	±0.01	0.78	±0.01	T *p* = 0.12
	+5 Weeks	0.77	±0.01	0.76	±0.01	0.75	±0.01	0.75	±0.01	Tr *p* = 0.13
	+24 Weeks	0.8	±0.01	0.78	±0.01	0.77	±0.01	0.78	±0.01	T × Tr *p* = 0.58

D2M (enhanced D2 mushroom), D3 (vitamin D3), SM (standard mushroom), and PL (placebo). Models controlled for baseline age, sex, MMSE, BMI, cohort and APOE status. *p*-values arising from mixed-effects regression models. T = time; Tr = treatment.

**Table 5:** Descriptive statistics (mean ±SE) for mood measures.

		D2M		D3		SM		PL		Model *p*
ANXIETY	Week 0	2.52	±0.26	3.1	±0.39	3.04	±0.34	3.13	±0.4	T *p* = 0.46
	+5 Weeks	2.77	±0.27	2.94	±0.4	2.57	±0.33	2.91	±0.4	Tr *p* = 0.86
	+24 Weeks	2.63	±0.29	3.53	±0.47	3.08	±0.39	2.87	±0.44	T × Tr *p* = 0.43
DEPRESSION	Week 0	4.31	±0.42	4.52	±0.59	4.44	±0.55	3.91	±0.47	T *p* = 0.17
	+5 Weeks	3.7	±0.39	4.18	±0.45	4.07	±0.41	3.54	±0.44	Tr *p* = 0.95
	+24 Weeks	4.06	±0.48	4.29	±0.52	4.26	±0.51	3.39	±0.42	T × Tr *p* = 0.97
STRESS	Week 0	7.46	±0.6	6.58	±0.55	7.93	±0.58	8.15	±0.61	T *p* = 0.04
	+5 Weeks	7.75	±0.59	7.21	±0.67	7.43	±0.54	7.7	±0.77	Tr *p* = 0.61
	+24 Weeks	7.14	±0.62	6.25	±0.61	7.11	±0.56	7.52	±0.59	T × Tr *p* = 0.92
POSITIVE AFFECT	Week 0	33.16	±0.91	34.53	±0.93	31.18	±0.84	34.57	±0.71	T *p* = 0.37
	+5 Weeks	32.7	±0.79	33.58	±1	31.66	±0.85	33.89	±0.74	Tr *p* = 0.12
	+24 Weeks	33.56	±0.8	34.73	±0.92	31.8	±0.92	35.43	±0.73	T × Tr *p* = 0.54
NEGATIVE AFFECT	Week 0	12.18	±0.49	11.1	±0.21	11.21	±0.28	11.68	±0.39	T *p* = 0.004
	+5 Weeks	11.89	±0.48	11.01	±0.17	10.92	±0.2	11.24	±0.29	Tr *p* = 0.06
	+24 Weeks	10.82	±0.18	11.13	±0.31	10.65	±0.13	11.4	±0.31	T × Tr *p* = 0.19
HAPPINESS	Week 0	5.66	±0.1	6.19	±0.52	6.14	±0.44	5.52	±0.1	T *p* = 0.29
	+5 Weeks	5.94	±0.16	5.57	±0.1	5.68	±0.11	5.61	±0.09	Tr *p* = 0.45
	+24 Weeks	5.92	±0.18	5.7	±0.09	5.73	±0.1	5.7	±0.08	T × Tr *p* = 0.45

D2M (enhanced D2 mushroom), D3 (vitamin D3), SM (standard mushroom), and PL (placebo). Models controlled for baseline age, sex, MMSE, BMI, cohort and APOE status. *p*-values arising from mixed-effects regression models. T = time; Tr = treatment.
